# Antitumor Effect and Immune Response of Nanosecond Pulsed Electric Fields in Pancreatic Cancer

**DOI:** 10.3389/fonc.2020.621092

**Published:** 2021-02-09

**Authors:** Jing Zhao, Shuochun Chen, Lu Zhu, Liang Zhang, Jingqi Liu, Danxia Xu, Guo Tian, Tian’an Jiang

**Affiliations:** ^1^ Department of Ultrasound, The First Affiliated Hospital, College of Medicine, Zhejiang University, Hangzhou, China; ^2^ Key Laboratory of Organ Transplantation, Research Center for Diagnosis and Treatment of Hepatobiliary Diseases, Hangzhou, China; ^3^ Division of Hepatobiliary and Pancreatic Surgery, Department of Surgery, The First Affiliated Hospital, Zhejiang University School of Medicine, Hangzhou, China

**Keywords:** nanosecond pulsed electric fields, pancreatic cancer, ablation, tumor microenvironment, immune response

## Abstract

Nanosecond pulsed electric fields (nsPEFs) have emerged as a novel and effective strategy for the non-surgical and minimally invasive removal of tumors. However, the effects of nsPEFs treatment on the tumor immune microenvironment remain unknown. In this study, the changes in the morphology and function of pancreatic cancer cells after nsPEFs were assessed and the modifications in the immune profile in pancreatic cancer models were investigated. To this end, electrodes were inserted with different parameters applied to ablate the targeted tumor tissues. Tumor development was found to be inhibited, with decreased volumes post-nsPEFs treatment compared with control tumors (P < 0.05). Hematoxylin and eosin staining showed morphological changes in pancreatic cancer cells, Ki-67 staining confirmed the effects of nsPEFs on tumor growth, and caspase-3 staining indicated that nsPEFs caused apoptosis in the early stages after treatment. Three days after nsPEFs, positron emission tomography demonstrated little residual metabolic activity compared with the control group. Gene expression profiling identified significant changes in immune-related pathways. After treatment with nsPEFs, CD8^+^ T lymphocytes increased. We showed that nsPEFs led to a significant decrease in immune suppressive cells, including myeloid derived suppressor cells, T regulatory cells, and tumor-associated macrophages. In addition, the levels of TNF-α and IL-1β increased (P < 0.05), while the level of IL-6 was decreased (P < 0.05). NsPEFs alleviated the immunosuppressive components in pancreatic cancer stroma, including hyaluronic acid and fibroblast activation protein-α. Our data demonstrate that tumor growth can be effectively inhibited by nsPEFs *in vivo*. NsPEFs significantly altered the infiltration of immune cells and triggered immune response.

## Introduction

Pancreatic cancer is one of the most virulent malignancies, with a rapid progression, a low rate of resectability, and an extremely poor prognosis. It is estimated that the 5-year overall survival rate for patients with pancreatic cancer is only about 3% (1). Approximately 15–20% of patients have the chance of surgical resection at the beginning of diagnosis, but only 20% of these survive for 5 years (2). The recommended treatment for unresectable pancreatic cancer is systemic chemotherapy or combined radiotherapy, although the median overall survival remains poor (6–12 months) (3). Current guidelines do not recommend surgery for patients with metastatic pancreatic cancer. However, local ablative strategies such as radiofrequency ablation, irreversible electroporation (IRE), and stereotactic body radiation have already gained their place as options to achieve disease control and long-term survival in addition to standard chemotherapy (4).

New local treatment based on electromagnetic fields is emerging as a promising strategy for tumors (5). High-voltage electric pulses cause permeable structures to form in the cell membrane (referred to “nanopores”), which affect all aspects of the cell physiology virtually (6). Reversible electroporation has been applied to increase the uptake of chemotherapy, thus inducing tumor regression. Irreversible electroporation creates permanent nanopores in the cell membrane, resulting in an imbalance of homeostasis and inducing the apoptosis of tumor cells. Significantly, the selective inactivation of the tumor without damaging important surrounding structures gives a unique advantage in the treatment of non-resectable pancreatic cancer (7). IRE is challenged by the risk of muscle contractions, and this procedure requires Electrocardiograph synchronization to avoid arrhythmia (8). Moreover, if the pulses are not sufficiently intense, cell viability can be preserved. The shorter the pulses, the higher the field strength (9). In contrast to IRE, the pulses of nsPEFs are ultrashort (10-300 ns), and vast amounts of energy can be released in a short time. Electrical pulses penetrate the organelle membrane, trigger caspase activation, increase the levels of calcium in the cytosol, and induce phosphatidylserine translocation (10). Data is increasingly indicating that nsPEFs are effective and minimally invasive methods for tumor ablation (11–13). However, there is little evidence of the effects of nsPEFs on pancreatic cancer. Moreover, whether it represents a potential cure for pancreatic cancer has yet to be elucidated, and no clinical imaging methods have been used to assess the therapeutic response of patients to nsPEFs. To this end, we developed a patient-derived tumor xenograft (PDX) model of pancreatic cancer and investigated the effectiveness of various nsPEFs parameters on the tumor. The histological features of tumors were evaluated post-nsPEFs. Positron emission tomography computed tomography (PET/CT) was performed to assess the early response to nsPEFs.

Accumulating evidence indicates that immune responses are essential for cancer development. Tumor therapy that is able to both ablate tumors minimally and stimulate the specific immune response to tumor cells will have a higher potential in clinical applications. However, whether nsPEFs exhibit inflammatory and immune-regulating effects are conflicting, and it is unclear whether nsPEFs can stimulate the antitumor effect of the immune system in pancreatic cancer with poor immunogenicity. Furthermore, little is known about the influence on cell signaling with short transient high-amplitude pulses, which are typically used in nsPEFs, for cancer treatment. In this study, we hypothesized that nsPEFs are able to stimulate the immune response in residual tumors and alleviate stroma-induced immunosuppression. Gene expression profiling identified significant changes in immune-related pathways. Our findings indicate that nsPEFs can promote the infiltration of CD8^+^ T cells and inhibit immunosuppressive cells. Our studies are also designed to explore the immune mechanism in nsPEFs treatment and provide new insights into immune therapy in combination with nsPEFs in cancer treatment.

## Materials and Methods

### Cell Line and Animals

All studies were performed under a protocol approved by the Zhejiang University Institutional Animal Care and Use Committee (Hangzhou, China). BALB/c nude mice^–^ (6 weeks old) were obtained from the Beijing Vital River Laboratory Animal Technology Co., Ltd. (Beijing, China). The mice (18–21 g) were housed in specific-pathogen-free surroundings with a 12 h light/dark cycle and *ad libitum* access to food and water. Fresh tumor samples were obtained from the operating room at the First Affiliated Hospital of Medical School of Zhejiang University. The tissue was immediately transferred onto ice in cold PBS solution (Hyclone Technologies, South Logan, UT, USA). Patient-derived tumors were then dissected, cut into fragments, and quickly grafted subcutaneously into the flank of nude mice. Panc02 cells were kindly provided by Stem Cell Bank, Chinese Academy of Sciences (Shanghai, China). The Panc02 pancreatic cancer model was established *via* the subcutaneous inoculation of 1 × 10^6^ Panc02 cells in 100 µl of Hanks balanced salt solution into the right flank of 6-week-old C57BL/6 mice. Tumor sizes were measured every 4 days after injection using a caliper. Tumor volume was calculated using the standard formula *V* = length × width× width/2. Twenty-seven nude mice were randomly divided into four groups with different electric field intensities: (1) group A (control group), in which seven mice were anesthetized and the electrode was placed on the tumor, but no nsPEFs were applied; (2) group B, in which seven mice were treated with nsPEFs of 30 kV/cm amplitude and 300 ns duration for 400 pulses in a single treatment; (3) group C, in which seven mice were treated with nsPEFs of 30 kV/cm amplitude and 300 ns duration for 200 pulses in a single treatment; (4) group D, in which six mice were treated with nsPEFs of 24 kV/cm amplitude and 300 ns duration for 400 pulses in a single treatment. Tumor volume was measured every 2 d. If the tumor volume exceeded 2 cm^3^, the mice were euthanized. The C57BL/6 mice with Panc02 tumors were treated with nsPEFs of 30 kV/cm amplitude, 300 ns duration for 400 pulses in a single treatment.

### NsPEFs Generator

**Figure 1:**
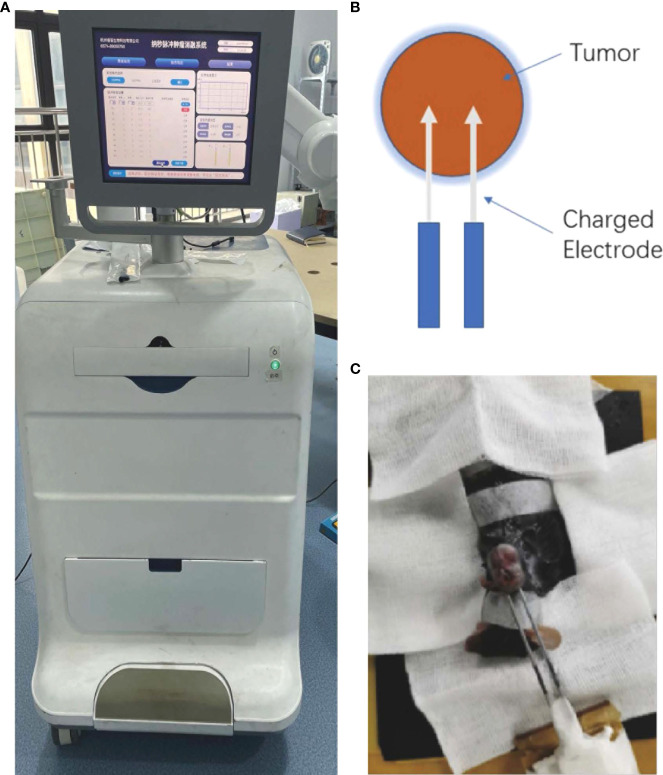
**(A)** Image of the nanosecond-pulsed tumor ablation system. **(B)** Schematic illustration of the treatment strategy. **(C)** Representative photograph showing nsPEFs electrode placement within the targeted tumor.

The application electrodes, pulse generator, voltage, and pulsing pattern of the nsPEFs were used as described previously (14). The dose effect was studied by varying the pulse numbers and the output voltage. The nsPEFs were delivered to subcutaneous tumors using a pair of electrodes, where the electrodes were placed on the periphery of the tumor. A semicircular electric field was formed between the two electrodes. The tumor was treated with a complete and uniform pulse field strength while avoiding the electrode directly through the air, resulting in electric spark discharge (**Figure 1**).

### NsPEFs Treatment Follow-Up

After nsPEF treatment, the mice were placed in their animal facility with free access to water and food. Tumor sizes were measured every 2 d.

### PET/CT Scanning

PET/CT images were acquired using an Albira small-animal PET/CT scanner (Bruker, Germany). PET/CT scanning was performed at the baseline and 3 d after nsPEFs to assess the effects of treatment. The mice in group B were anesthetized and fluorine 18 fluorodeoxyglucose (18F-FDG) was administered *via* the tail vein at a dose of 3.70 to 4.44 MBq in 0.1–0.2 ml of saline 60 min prior to imaging (15). PET scans were conducted for 15 min, and the CT scan for 5 min was performed. The standardized uptake value of the tumor and organs was measured as the percentage of injected radioactivity dose/gram (% ID/g) using the Albira PET system and PMOD (version 3.7) software (PMOD Technologies, Switzerland).

### Immunochemistry

Sixteen mice were treated with nsPEFs of 30 kV/cm amplitude, 300 ns duration for 400 pulses in a single treatment.

At various intervals after nsPEFs treatment (0, 1, 3, and 14 d), sixteen nude mice previously grafted with human tumor were euthanized by cervical dislocation and the tumors were excised. The harvested tissues were fixed rapidly in 4% paraformaldehyde, embedded in paraffin, and sliced into 5-µm sections. Some slices were stained with hematoxylin and eosin, dehydrated using an ethanol gradient (100%, 90%, and 75%), and deparaffinized with xylene. Some of these slices were immunostained using antibodies against caspase-3 (Biocare Medical, Concord, CA) for the analysis of apoptotic cell death. Ki-67 staining was used as a proliferation marker in malignant tumors. Stroma within the tumor was also detected with α-smooth muscle actin (α-SMA) (Affinity Biosciences, Cincinnati, USA), fibroblast activation protein-α (FAP-α) (Affinity Biosciences, Cincinnati, USA), and hyaluronan-binding protein 1 (HABP1) (Affinity Biosciences, Cincinnati, USA) staining.

### RNA-Seq Experiment

Total RNA was extracted using a mirVana miRNA isolation kit (Ambion) following the manufacturer’s protocol. RNA integrity was evaluated using an Agilent 2100 Bioanalyzer (Agilent Technologies, Santa Clara, CA, USA). Samples with RNA Integrity Number (RIN) ≥ 7 were subjected to subsequent analysis. The libraries were constructed using TruSeq Stranded mRNA LT Sample Prep kit (Illumina, San Diego, CA, USA) according to the manufacturer’s instructions. These libraries were then sequenced on an Illumina sequencing platform (HiSeq TM 2500 or Illumina HiSeq X Ten) and 125 bp/150 bp paired-end reads were generated. Hierarchical cluster analysis of the differentially expressed genes (DEGs) was performed to determine the gene expression patterns. gene ontology (GO) enrichment and KEGG pathway enrichment analysis of DEGs were performed using R based on the hypergeometric distribution.

### Flow Cytometric Analysis

Sections of the spleen, axillary lymph nodes, and tumors were prepared as single cell suspensions. The following flow cytometry antibodies were use: anti-mouse CD3 PE-Cyanine5 (15-0031-81; eBioscience), anti-mouse CD4 PE(12-0041-81; eBioscience), anti-mouse CD 8 FITC (12-0081-81; eBioscience), anti-mouse LY-6C APC (17-5932-80; eBioscience), anti-mouse LY-6G FITC (11-9886-80; eBioscience), anti-mouse F4/80 PE (565410; BD Pharmingen), anti-mouse CD11b APC-Cy7 (561039; BD Pharmingen), anti-mouse CD4 PE-Cyanine5 (15-0041-81; eBioscience), anti-mouse CD25 PE (12-0251-81; eBioscience), anti-mouse Foxp3 Alexa Fluor^®^ 488 (126405; Biolegend). Intracellular staining was performed after conducting fixation and permeabilization using Transcription Factor Buffer Set (424401; Biolegend). Cells were incubated on ice for 30 min before analyzed the samples using a FACS Canto II cytometer. The resulting data were processed using FlowJo (version 10.0.7) software.

### Assessment of Supernatant Protein Concentration Using ELISA

To measure the circulating TNF-α, IL-6, and IL-1β levels *in vivo*, blood was removed from the eyes. The samples were stored frozen before sample testing. The levels of TNF-α, IL-6, and IL-1β in the murine serum samples were analyzed using TNF-α ELISA kit (70-EK282HS-96; MULTI SCIENCES), IL-6 ELISA kit (70-EK206HS-96; MULTI SCIENCES), and IL-1β ELISA kit (EK201B/3-96; MULTI SCIENCES) according to the manufacturer’s instructions.

### Assessment of Chemokines in Tumor Using ELISA

To demonstrate the presence of chemokines able to attract T cells in the tumor, tumor samples were removed from the mice. The samples were stored frozen from sample testing. The supernatant was taken for testing after homogenizing the tumor tissue. The level of CCL2, CXCL9 in the tumor samples were analyzed using CCL2 ELISA kit (EK287/2-48, MULTI SCIENCES), CXCL9 ELISA kit (EK2143/2-96, MULTI SCIENCES) according to manufacturer’s instructions.

### Statistical Analysis

All data are presented as the mean ± standard deviation (SD). Statistical analysis was performed using GraphPad Prism (version 5.0) (USA). Survival rates in different groups were evaluated using the Kaplan-Meier survival method and log-rank test. Student’s t-test was used to analyze paired groups, and one-way analysis of variance (ANOVA) was used to analyze multiple groups. P < 0.05 was considered significant.

## Results

### NsPEFs Treatment Inhibited Tumor Growth in PDX Models of Pancreatic Cancer

**Figure 2:**
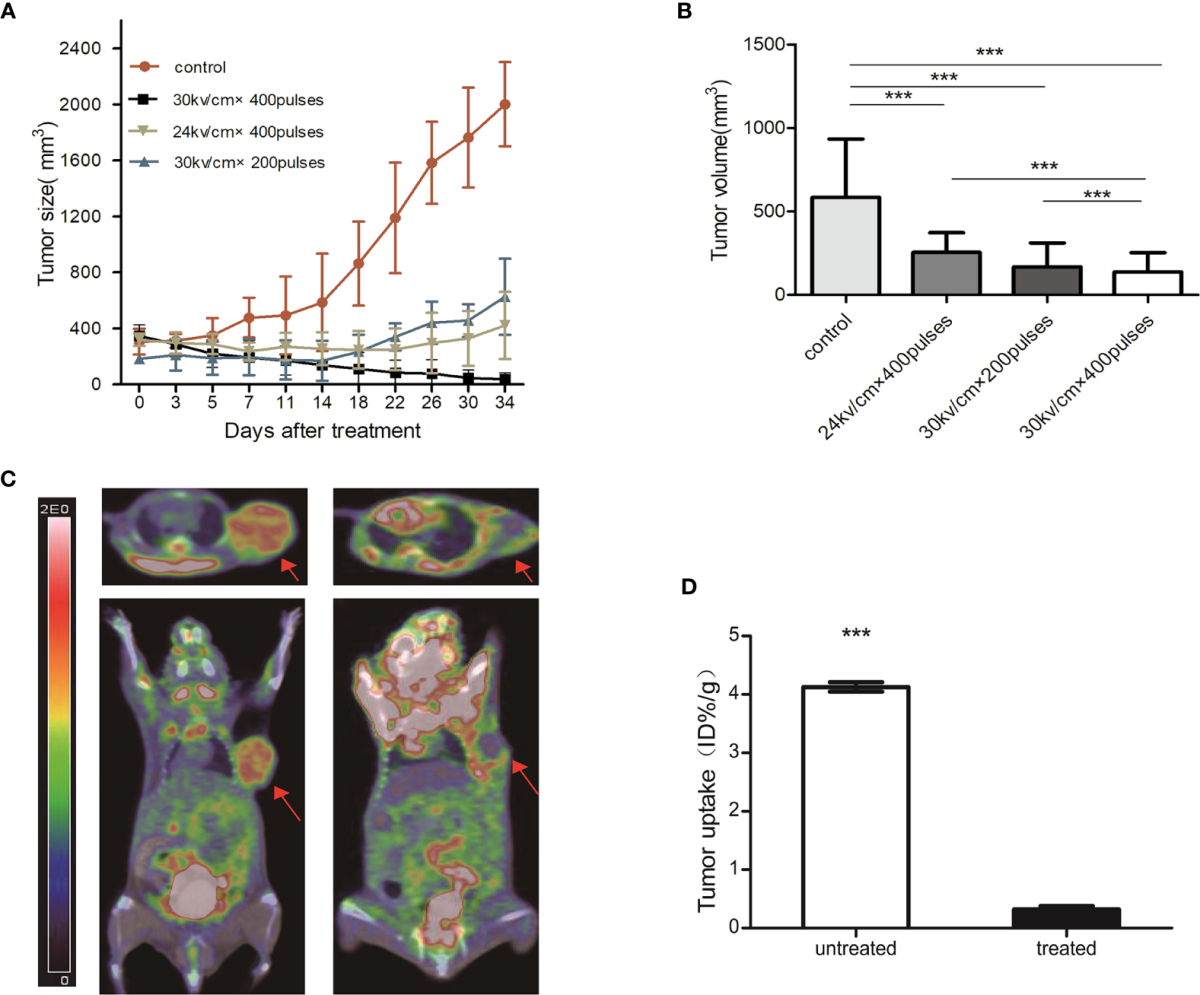
**(A)** Growth curve of pancreatic cancer tumor from PDX models post-nsPEFs treatment. Tumor volume was determined using calipers after treatment with different parameters. **(B)** Tumor volume comparison 14 days after treatment. The tumor volume of treated mice decreased significantly compared with control which had no nanosecond pulsed electric fields treatment (***p < 0.001). **(C)** PET/CT scans obtained at baseline and 3 days after nsPEFs treatment. Tumors had substantial initial FDG activity on the baseline fused FDG PET/CT images. FDG PET/CT scans obtained after nsPEFs showed limited metabolic activity. **(D)** Bar chart showing a significant difference in tumor uptake (ID%/g) between treated and untreated tumors 3 days after treatment (***p < 0.001).

At the time of nsPEFs treatment, the tumor reached an average volume of 400 mm^3^ 40 d after the graft. Mice were divided into four groups according to the different treatments. Tumor growth was inhibited by nsPEFs treatment. Control tumors in all mice grew faster and larger than the treated tumors (**Figure 2A**). Group B achieved a statistically significant reduction in tumor volume compared to the other groups on day 32 (**Figure 2A**). All treatment groups achieved a statistically significant decrease in tumor volume compared with the control group 14 d after treatment (**Figure 2B**). Tumors in the control group (group A) were more than 1.9 times in size by day 32. Group B had 3 complete regressions, while the other tumor measured 66.7 mm^3^ on day 32. In group C and D, tumor growth was markedly slower than that of the control group, with tumors measuring 629.6 and 421.5 mm^3^ on day 32.

### Metabolic Information Provided by PET/CT

All tumors were successfully visualized using PET/CT, with scans acquired at the baseline and 3 d after nsPEFs (**Figure 2C**). The tumor mass was identified on CT images. Three days after nsPEFs, the mean tumor uptake obtained from the regions of interest was 0.3 ID%/g (n = 5) for nsPEFs-treated tumors and 4.1 ID%/g for untreated tumors (n = 5). The differences between treated and untreated tumors were significant (P < 0.001) (**Figure 2D**).

### NsPEFs Induced Cell Necrosis and Apoptosis and Inhibited Tumor Growth *In Vivo*

**Figure 3:**
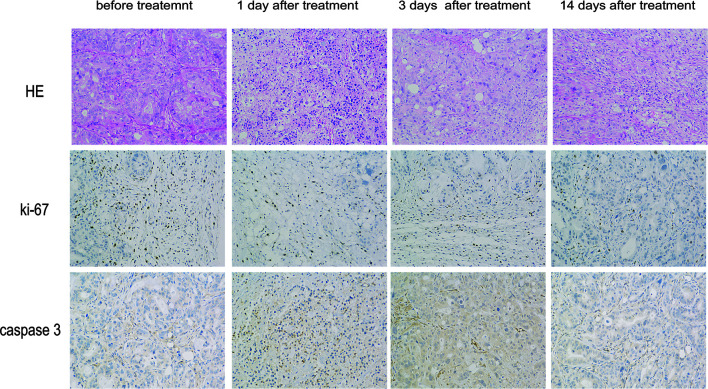
Histopathology of pancreatic cancers after nsPEFs treatment. 0, 1, 3, and 14 days after treatment, the mice were euthanized and samples were harvested. Tumor cell structure and nuclear changes were analyzed by hematoxylin and eosin (H&E) staining. Samples were also prepared for immunohistochemistry with antibodies to Ki-67 and caspase-3. Magnification, 200×.

In the pathological examinations, necrosis was not observed in the control tumors, whereas large areas of necrotic tissue were found inside the other tumors. The parenchymal component cells were arranged loosely, and a large number of cell fragments were visible in the treated group, which was most notable 1 d after treatment. Fourteen days after treatment, the parenchymal component cells were found to be arranged in a disorderly manner in the treatment group. In addition, Ki-67 staining in the treatment group was significantly lower than that in the control group, which decreased gradually over time. To verify whether nsPEFs can decrease pancreatic cancer growth by promoting tumor cell apoptosis *in vivo*, we examined the markers related to cell apoptosis using immunohistochemistry. Caspase-3 staining showed that the proportion of positive cells after treatment was significantly higher than that of the control group (**Figure 3**). A large number of tumor cells undergo necrosis and apoptosis in the early stage after treatment, which can explain the PET/CT imaging results.

### NsPEFs Significantly Alters Gene Expression Profiles in Immune Signaling Pathways

**Figure 4:**
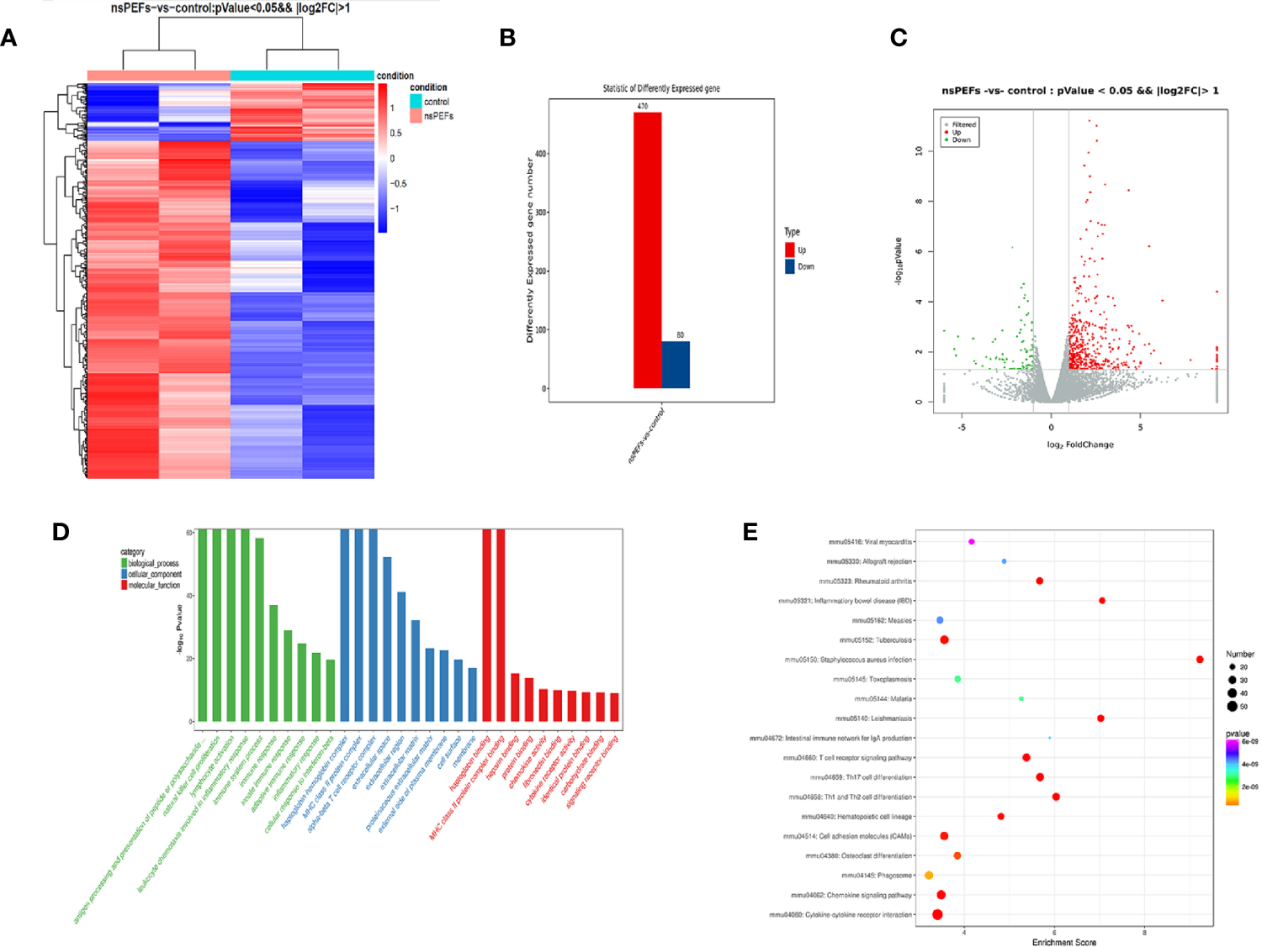
**(A)** Hierarchical clustering of differentially expressed mRNAs. Hierarchical clustering was performed using differentially expressed mRNAs between the nsPEFs-treated group and the control group. The gene expression values (log2-transformed intensities) are scaled and depicted in color code format (upregulated: red, downregulated: blue). **(B)** Bar chart of differentially expressed mRNAs between the nsPEFs-treated group and the control group (upregulated: red, downregulated: blue). **(C)** The expression profiles of the identified differentially expressed genes (DEGs). Red and green points represent the significant DEGs with *p* < 0.05 and log2(fold change) >1, and grey points show those without significance, respectively. Fold change refers to the values of FPKM change. **(D)** GO classification of DEGs. GO terms are summarized in three main categories: cellular component, molecular function, and biological process. **(E)** Top 20 pathways of KEGG functional enrichment among DEGs. The color of nodes changes from purple - blue - green - red, and the smaller the enrichment p-value, the greater the significance. The point size denotes the DEG number.

To obtain an unbiased understanding of the underlying molecular mechanism of nsPEFs, we performed RNA-seq analysis using fresh residual Panc02 tumors 3 d after nsPEFs, as well as control tumors. A total of approximately 60 million 100 base-paired-end reads were obtained per sample. Numerous significant changes were found in multiple genes and pathways between the nsPEFs-treated tumor and the untreated tumor (**Figures 4A, C**). Among 550 DEGs, 470 genes were found to be upregulated and 80 genes downregulated in the nsPEFs group compared with the control group (**Figure 4B**). Our data showed that nsPEFs influenced multiple populations of immune cells within the tumor microenvironment. GO enrichment analysis demonstrated an increase in gene expression profiles, suggesting that “antigen processing and presentation of peptide or polysaccharide antigen *via* MHC class II; MHC class II protein complex” showed an increasing trend in the treatment groups (**Figure 4D**). Then, whether certain KEGG pathways were enriched was analyzed. Interestingly, some immune-related pathways, such as Th1 and Th2 cell differentiation, TGF-β signaling pathway, inflammation bowel disease (IBD), and cytokine-cytokine receptor interaction, were found to be affected by nsPEFs (**Figure 4E**). Future studies will need to evaluate gene expression at the single-cell level to work around the inherent biological variability associated with animal studies.

### T Cell Subset Changes After nsPEFs Treatment for Pancreatic Cancer

**Figure 5:**
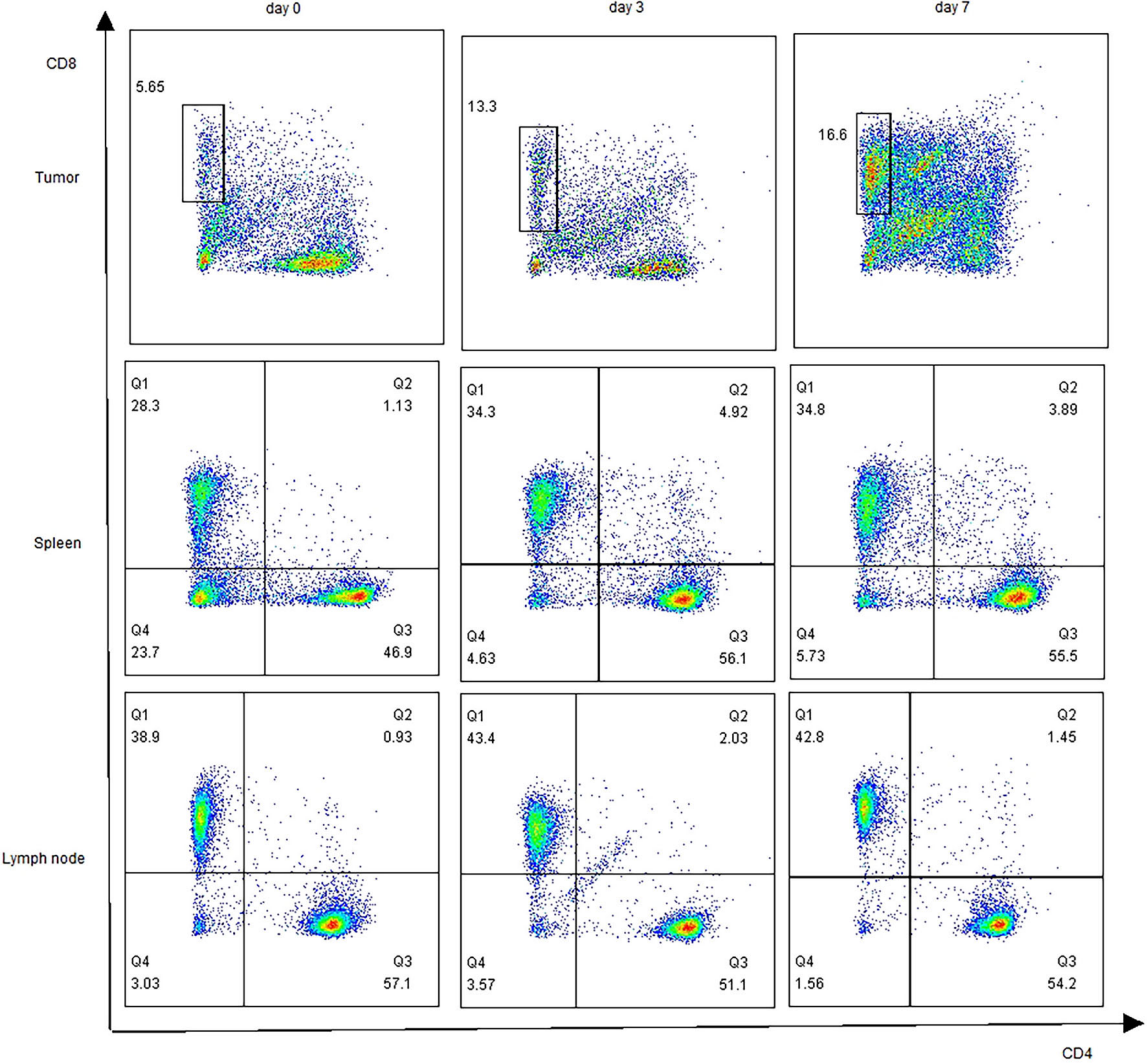
Profiling of T cells after nsPEFs treatment. The ratios of CD4^+/^CD3^+^, CD8^+^/CD3^+^in response to nsPEFs treatment in the tumor tissue, spleen, and axillary lymph nodes on day 3 and 7. One representative flow cytometric graph for each pattern was shown here (n = 4). Data are presented as the mean ± SD. P-values were calculated based on a Student’s t-test (n = 4 per group).

To determine whether nsPEFs induced an immune response after the treatment of pancreatic cancer, we analyzed the infiltrating immune cells in the residual tumor, spleen, and lymph nodes in comparison to those in untreated tumors at an earlier (day 3) and later time point (day 7) in the Panc02 tumor models. We observed the ratio of CD8^+^ T cells was increased in nsPEFs-treated mice (**Figure 5**). Compared with the control group, CD8^/^CD3 ratios in the tumor was significantly elevated (2.01-fold, day 7). In addition, CD8^/^CD3 ratios in the spleen and axillary lymph node exhibited slight increases (1.39-fold and 1.09-fold on day 7, respectively). CD4^/^CD3 ratios in the spleen cells is strongly increased 3 days and 7 days after the treatment in the spleen (1.12-fold and 1.25-fold).

### NsPEFs Alters the Immune Microenvironment, Rendering It Less Immune Suppressive

**Figure 6:**
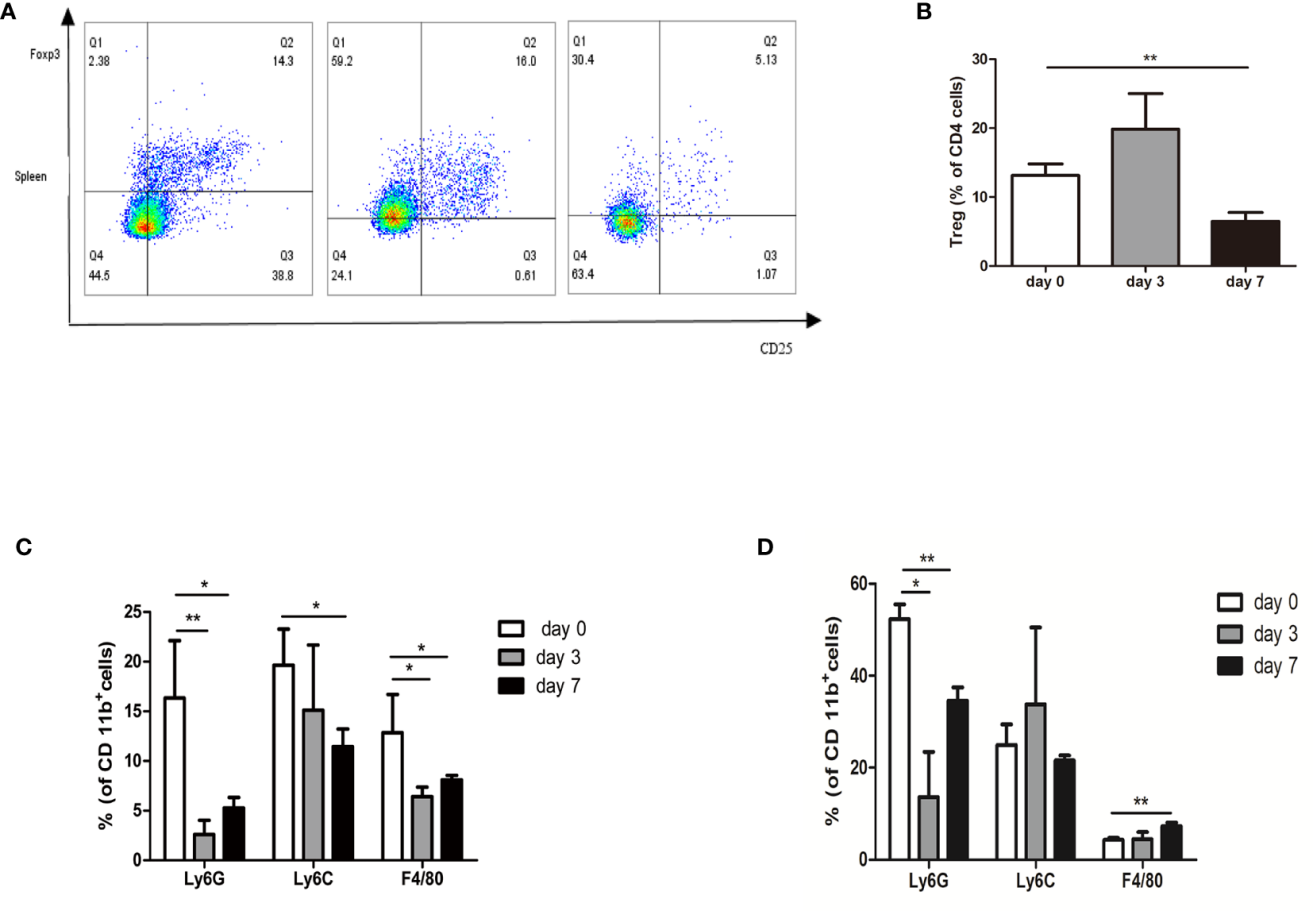
Profiling of immune suppressive cells after nsPEFs treatment. **(A)** Frequency of Treg cells in response to nsPEFs treatment in the spleen on day 3 and 7. Analyses were performed on CD4^+^ cells. **(B)** Frequency of MDSCs (CD11b^+^Ly6G^+^ and CD11b^+^Ly6C^+^) and macrophages (CD11b^+^F4/80^+^) in the tumor on day 3 and 7. **(C)** Frequency of MDSC (CD11b^+^Ly6G^+^ and CD11b^+^Ly6C^+^) and macrophages (CD11b^+^F4/80^+^) in the spleen on day 3 and 7. **(D)** Frequency of MDSC (CD11b^+^Ly6G^+^ and CD11b^+^Ly6C^+^) and macrophages (CD11b^+^F4/80^+^) in the tumor on day 3 and 7. Data are presented as the mean ± SD. P-values were calculated based on a Student’s *t*-test (n = 4 per group). *p < 0.05, **p < 0.01.

**Figure 6A** shows the changes in Tregs in the spleen after treatment. A decrease in Treg cell infiltration was observed in the spleen on day 7 (6.49%), although a slight increase in Treg cells was observed on day 3 (19.87%) (**Figure 6B**). Myeloid cells were subdivided into neutrophilic (CD11b^+^Ly6G^hi^) myeloid derived suppressor cells (nMDSC), monocytic (CD11b^+^Ly6C^hi^) myeloid derived suppressor cells (mMDSC), and macrophage cells (CD11b^+^F4/80^+^). MDSC subsets showed a decrease in the percentage of CD11b^+^ cells, where the decrease in neutrophilic type (nMDSC) was the largest in the tumor (**Figure 6C**). Simultaneously, we observed that the percentage of nMDSCs and mMDSCs was decreased in the spleen. Interestingly, we observed a slight increase in macrophage cell infiltration in the spleen on day 7 (7.28% vs. 4.33%) (**Figure 6D**).

### The Anti-Tumor Effect of nsPEFs-Treated Mice Is Related to the Level of Immune Cytokines and Chemokines

**Figure 7:**
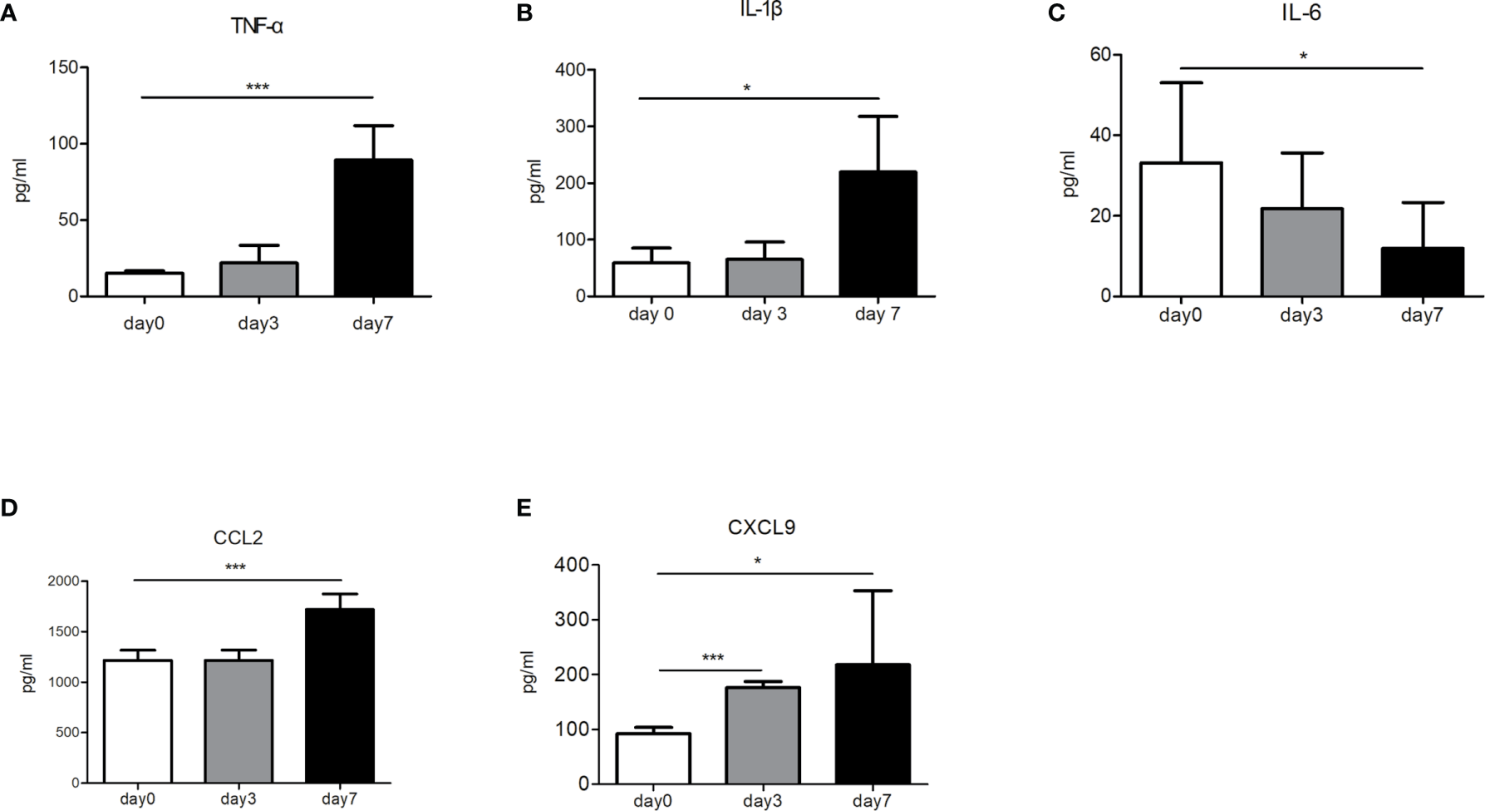
The concentrations of immune cytokines and chemokines before and after nsPEFs treatment. **(A)** Expression levels of TNF-α in the blood. **(B)** Expression levels of IL-1β in the blood. **(C)** Expression levels of IL-6 in the blood. **(D)** Expression levels of CCL2 in the tumor. **(E)** Expression levels of CXCL9 in the tumor. Data are presented as the mean ± SD. P-values were calculated based on a Student’s t-test (n>3 independent experiments). *p < 0.05, ***p < 0.001.

To elucidate the mechanisms underlying the immune reactions, we analyzed the immune factors of tumor-bearing mice (control group vs. nsPEFs treatment group). After nsPEFs treatment, the levels of TNF-α and IL-1β were found to increase (P < 0.001 and P < 0.05, respectively) (**Figures 7A, B**), whereas the content of IL-6 was reduced (P < 0.05) (**Figure 7C**). The levels of CCL2 and CXCL 9 were found to increased aftet nsPEFs treatment (**Figures 7D, E**). The experimental results suggest that the immune responses of nsPEFs treatment may be related to the levels of immune cytokines and chemokines.

### NsPEFs Induced a Modulation of Stroma

**Figure 8:**
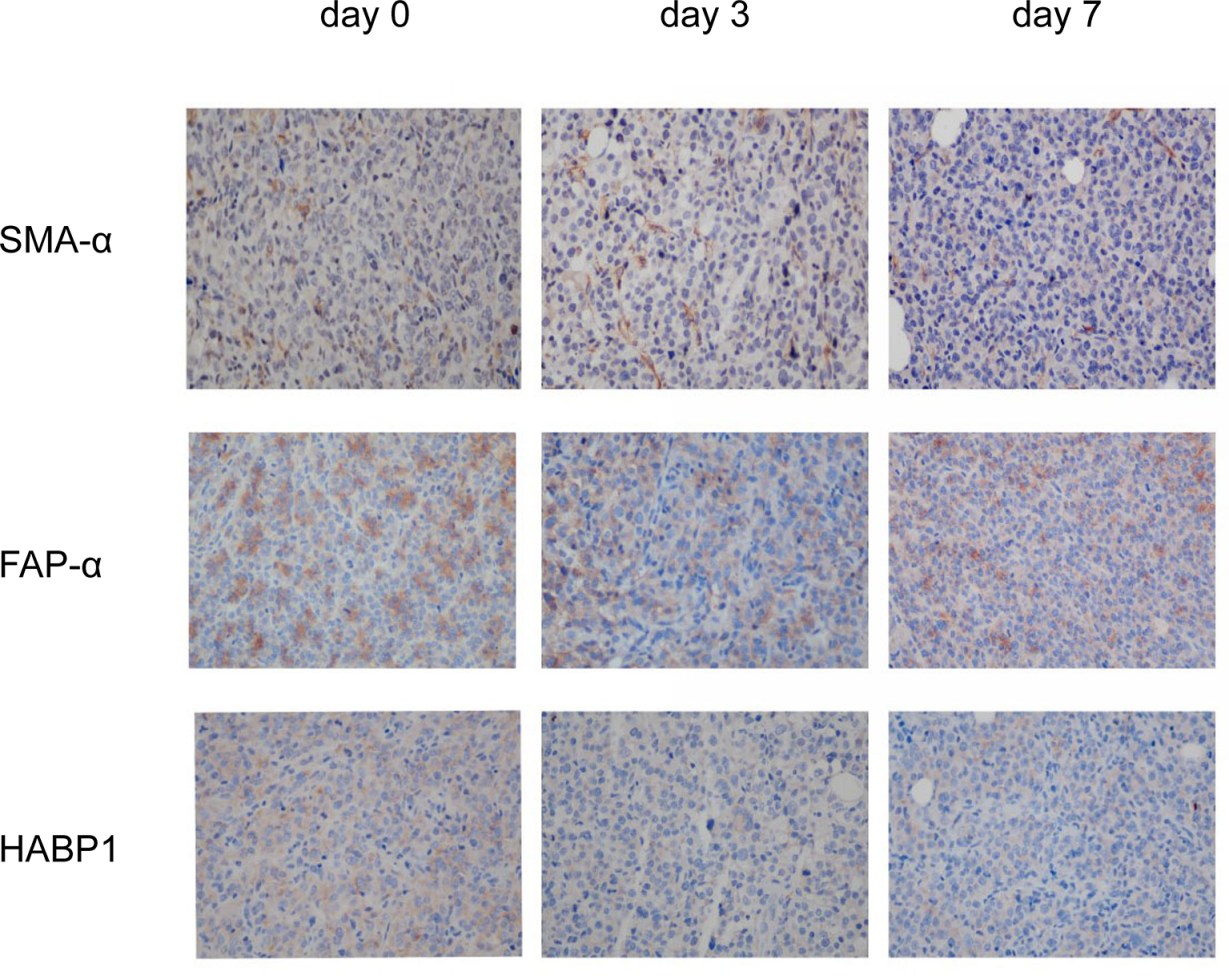
IHC staining of viable tumor region at 3 and 7 days after the initiation of treatment. Representative micrographs of staining for α-SMA, FAP-α, HABP1. Five visual fields were randomly captured for each group. Magnification, 200×.

It is well known that the modulation of pancreatic cancer stroma enhances the tumor infiltration of CD8^+^ cells. Since the preliminary results suggested that nsPEFs can increase the infiltration of CD8^+^ T cells in tumors, next, we investigated the effect of nsPEFs on tumor stromal components. The expression levels of α-SMA in the treatment group were the same as those in the control group. The expression of FAP-α and HABP1 was mostly reduced after nsPEFs treatment (**Figure 8**). These findings indicate that nsPEFs can modulate tumor stroma by softening the extracellular matrix (as indicated by reduced FAP-α and the depletion of hyaluronic acid), all of which favor tumor infiltration by T lymphocytes.

## Discussion

The present study is the first to establish a PDX model of pancreatic cancer to evaluate the effect of nsPEFs with different parameters. The *in vivo* response may be more robust for its translation to the clinical applications than conventional cell line-based xenografts. PDX models provide additional material to examine the biology of pancreatic cancer, and serve as a robust and preclinical model to examine the efficacy of this potential new therapy. Our results showed that the differences in dose effect for nsPEFs and the number of pulses and voltage are all significant factors that influence the effect of this treatment. All mice treated with nsPEFs tolerated the therapy well. Remarkably, we found that the treatment group with 30 kV/cm and 400 pulses experienced a significant reduction in tumor volume compared to the untreated controls. Imaging-based approaches for early postprocedural monitoring are critical for optimizing nsPEFs treatment. Our study showed that 18F-FDG can detect early metabolic response to nsPEFs treatment, which has not previously been well studied in the literature. In addition, our work provides a new insight into the molecular mechanisms underlying the role of nsPEFs *via* transcriptome sequencing. New adjuvant treatments that induce anti-tumor immunological effects have shown great potential for tumor destruction. In recent years, the number of studies on immunotherapy for pancreatic cancer has been increasing, which may open up new avenues for treatment. Whether nsPEFs can induce an anti-tumor immunological effect is controversial in pancreatic cancer. However, our results demonstrated that nsPEFs are able to address the lack of immunogenicity and the immunosuppressive tumor microenvironment (TME) of pancreatic cancer. Further mechanistic studies revealed that the immunomodulatory effect can be attributed to the secretion of cytokines and the alleviation of immunosuppressive components in pancreatic cancer stroma, including FAP-α and hyaluronic acid.

Despite the advances made over the years, the detailed mechanisms underlying the action of nsPEFs have yet to be fully elucidated. In the present study, a comprehensive view of the cellular dynamics induced by nsPEFs in pancreatic cancer tumors was presented. As a result, several signaling pathways related to inflammatory response were found to be altered in response to nsPEFs, consistent with the results of subsequent experiments. Our study also demonstrated that the granzyme-mediated apoptotic signaling pathway, cytolysis, apoptosis, and MAPK signaling cascades were involved, providing novel insights into the molecular mechanisms underlying the action of nsPEFs.

Cancer immunotherapy is emerging as a promising method for tumor therapy owing to its remarkable efficacy and innovation (16). However, the effects of nsPEFs on immune status are unclear. To counteract this, several studies on the immune response induced by nanosecond pulses in different types of tumors have been conducted. In one study, Beebe et al. showed that nsPEFs (KV/cm, ns, 1 Hz) not only ablated 90% of N1-S1 HCC tumors but also induced an immuno-protective effect in 100% of animals, defending animals against recurrences (17, 18). In another study, Jing Wang showed that a nanosecond pulsed electric field inhibited malignant melanoma growth by inducing a change in the systemic immunity (30 kV/cm, 100 ns, and 200 p) (19). However, Mollica and Muratori’s study suggested that nsPEFs did not boost the natural antitumor immunity that remains dormant in B16F10 melanoma tumors (750 p, 200 ns, 25 kV/cm, and 2 Hz). However, these studies used different electric pulse parameters and tumor models, which may help to explain the difference in their conclusions. Our data provide several new findings to the preliminary data available in the existing literature. Currently, only one article has addressed changes in the immune profile following nsPEFs treatment in pancreatic cancer: Beebe et al. found that the numbers of both Tregs and MDSCs were markedly reduced in the blood but not in the spleen or tumors. In addition, the number of dendritic cells in the TME was increased and multiple activation markers were upregulated following treatment. In general, the treatment of pancreatic tumors with nsPEFs (200 ns, 2 Hz, and 30 kV/cm) is effective, but further optimization is needed to induce a stronger immune response. In the present study, different electric pulse parameters (300 ns, 4 Hz, and 30 kV/cm) were used, which may influence the immunogenicity of treatment with nsPEFs. In our study, nsPEFs increased the infiltration of CD8^+^ cells, and the CD8^+^/CD3^+^ ratio was significantly higher than that in the control group 7 d in the TME after treatment. We also observed CD8^+^ T cell infiltration in the spleen and lymph nodes, indicating that nsPEFs induced T cell response. In pancreatic cancer, the infiltration of immune suppressive cells, including Tregs, MDSCs, and macrophages, makes the environment present as an immunosuppressive phenotype (20). MDSCs are generated from the bone marrow and are activated to suppress non-specific immune responses. It has also been well established that MDSCs are able to inhibit the T cell immune response and delay tumor progression (19). Tregs are a heterogeneous subset of immunosuppressive T cells that silence anti-tumor immune surveillance (21). Elevated levels of circulating myeloid-derived suppressor cells (MDSCs) and regulatory T cells (Tregs) are associated with a worse prognosis and overall survival (OS) in patients with pancreatic cancer (22). Stromal macrophages limit CD8^+^ T cell infiltration and migration (23). In the present study, large panels of antibodies were used to study the main populations of myeloid cells (nMDSC, mMDSC, and TAM). In contrast to Beebe et al., Tregs were found to be clearly reduced in the spleen. The subset of MSDCs and macrophages in the TME were also decreased compared with the control group. In the spleen, the frequency of nMDSCs decreased, whereas that of macrophages slightly increased 7 d after treatment. These findings suggest that nsPEFs (300 ns, 4 Hz, and 30kV/cm) inhibit immunosuppressive cells, which could partially explain the increase in T cells after nsPEFs treatment. Thus, nsPEFs treatment changed the immunosuppressive sequelae in the TME, accompanied by variable increases in CD8^+^ T cells in the tumor, spleen, and lymph nodes.

The dynamic and continuous inflammatory responses are intimately related to cytokines produced by tumor or inflammatory cells. Some cytokines have a great influence on cancer immunoediting and cancer progression. In our study, we evaluated the changes in several important pro-inflammatory cytokines in pancreatic cancer, including TNF-α, IL-6, and IL-1β. In our studies, the levels of TNF-α and IL-1β were found to increase after treatment. TNF-α is the master mediator of the inflammatory and immunologic response, inducing the infiltration of leukocytes and increasing the production of other cytokines and chemokines. The combination of cytokines and chemokines may contribute to the action of inflammatory cells, thereby reshaping the microenvironment (24). In addition to TNF-α, IL-1β is a prototypical proinflammatory cytokine that stimulates both local and systemic responses. The production of TNF-α and IL-1β at the inflammatory site is most likely triggered by cellular contact with stimulated infiltrating T cells (25). IL-6 seems to play an important role in pancreatic cancer, with several studies indicating that high levels of IL-6 expression are associated with a significantly lower survival and a poor response to therapy (26–28). Therefore, the IL-6 signaling pathway may serve as a promising therapeutic target for pancreatic cancer (29, 30). Our results have revealed that nsPEFs substantially inhibit the secretion of IL-6, which can limit T lymphocyte-driven antitumor immunity by reducing immune suppressive cells, such as myeloid-derived suppressor cells and regulatory T cells (Treg). In addition, nsPEFs may inhibit the invasion of pancreatic cancer cells by downregulating IL-6 expression, although a detailed mechanism for this is yet to be elucidated. The extremely strong immunosuppressive TME is a hallmark of pancreatic cancer, with little lymphocyte infiltration and increased immunosuppressor cells. The desmoplastic stroma of pancreatic cancer may be related to its immune evasion (31). The immunosuppressive TME was modulated by nsPEFs. Several components of fibrotic stroma were downregulated, including FAP-α and hyaluronic acid (indicated by the levels of HABP1 expression). All of these factors can limit the infiltration of T lymphocytes in pancreatic cancer. FAP is a transmembrane serine protease that is highly expressed in the cancer-associated stromal cells of epithelial cancers. FAP-α expressing cells are a significant immunosuppressive component that can result in the hypoxic necrosis of tumor and stroma cells through a process involving IFN-γ and TNF-α. Depleting FAP-expressing cells allowed for immunological-controlled growth in pancreatic cancer (32). Hyaluronan-binding protein 1 (HABP1) acts as a plasmalemmal receptor for C1q protein, which plays an important role in inflammatory responses and regulates cell adhesion, tumor invasion, tumorigenesis, and progression. HABP1 overexpression is associated with tumor malignancy and patient survival (33). In our study, SMA expression was not affected by nsPEFs treatment. Decreased α-SMA is correlated with poor prognosis and overall survival in patients with pancreatic cancer (34). Recently, studies have revealed that the depletion of α-SMA augmented pancreatic cancer and diminished overall survival (35). The preservation of α-SMA ^+^CAFs may have prevented the tumor from unchecked growth and prolonged the survival time of the mice. In addition, the modulation of the stroma may influence the secretion of cytokines. IL-6 is produced mainly by pancreatic stellate cells and tumor-associated myeloid cells, with some studies pointing out that pancreatic stroma is a source of IL-6 (36). Our experimental results suggested that the levels of cytokines may be related to the modulation of the stroma. Taken together, this study if the first to present two aspects of the effect of changes in immunity. Firstly, nsPEFs were found to influence the production of inflammatory-related cytokines, and secondly, nsPEFs were found to suppress desmoplastic changes in tumors, the latter of which influence the infiltration of immune cells. Further studies of the desmoplastic reaction will be needed to confirm these results.

This study has two noteworthy limitations. Firstly, only the early immune response (day 3 and 7) was evaluated. More information from different time points will be needed in order to fully evaluate the cellular response. Secondly, the impact of nsPEFs on inflammation cells will need to be explored further using functional tests in order to link these phenotypic variations with the capabilities.

To conclude, nsPEFs were found to significantly suppress the growth of pancreatic cancer. The findings presented in this study indicate that NsPEF ablation is able to trigger immune response by increasing T cell infiltration and decreasing immune suppressive cells. Given the immune-active nature of nsPEFs, the combined use of nsPEFs and immunotherapy in pancreatic cancer is worthy of consideration in future clinical applications.

## Data Availability Statement

The datasets presented in this study can be found in online repositories. The names of the repository/repositories and accession number(s) can be found in the article/supplementary material.

## Ethics Statement

The animal study was reviewed and approved by The Tab of Animal Experimental Ethical Inspection of the First Affiliated Hospital, College of Medicine, Zhejiang University.

## Author Contributions

JZ and SC performed the experiments and wrote the manuscript. LZ, LAZ, and DX contributed to perform the experiments. JL and GT contributed to interpret the data. TJ designed the experiments and analyzed and interpreted the data. All authors contributed to the article and approved the submitted version.

## Funding

This work was supported by the Nation Natural Science Foundation of China (no. 81971623) and Development Project of National Major Scientific Research Instrument (82027803).

## Conflict of Interest

The authors declare that the research was conducted in the absence of any commercial or financial relationships that could be construed as a potential conflict of interest.
