# Corrigendum: Antitumor effect and immune response of nanosecond pulsed electric fields in pancreatic cancer

**DOI:** 10.3389/fonc.2022.1052763

**Published:** 2022-10-11

**Authors:** Jing Zhao, Shuochun Chen, Lu Zhu, Liang Zhang, Jingqi Liu, Danxia Xu, Guo Tian, Tian’an Jiang

**Affiliations:** ^1^ Department of Ultrasound, The First Affiliated Hospital, College of Medicine, Zhejiang University, Hangzhou, China; ^2^ Key Laboratory of Organ Transplantation, Research Center for Diagnosis and Treatment of Hepatobiliary Diseases, Hangzhou, China; ^3^ Division of Hepatobiliary and Pancreatic Surgery, Department of Surgery, The First Affiliated Hospital, Zhejiang University School of Medicine, Hangzhou, China

**Keywords:** nanosecond pulsed electric fields, pancreatic cancer, ablation, tumor microenvironment, immune response

In the published article, there was an error in the legend for **Figure 1A** as published. Due to copyright reasons we want to change **Figure 1A** to the picture of the same machine taken by ourselves. The corrected **Figure 1** appears below.

**Figure 1:**
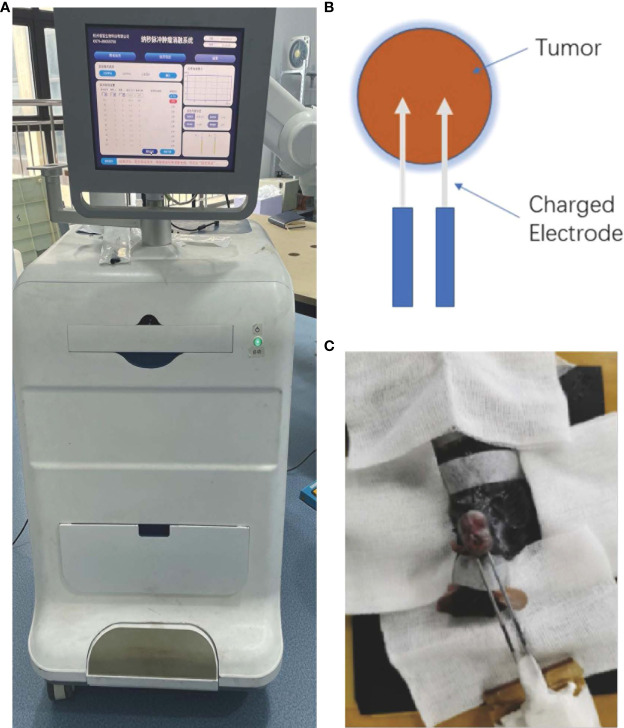
**(A)** Image of the nanosecond-pulsed tumor ablation system. **(B)** Schematic illustration of the treatment strategy. **(C)** Representative photograph showing nsPEFs electrode placement within the targeted tumor.

The authors apologize for this error and state that this does not change the scientific conclusions of the article in any way. The original article has been updated.

## Publisher’s note

All claims expressed in this article are solely those of the authors and do not necessarily represent those of their affiliated organizations, or those of the publisher, the editors and the reviewers. Any product that may be evaluated in this article, or claim that may be made by its manufacturer, is not guaranteed or endorsed by the publisher.

